# Impact of Rhegmatogenous Retinal Detachment Repair on Macular Microvascular Changes Detected by Optical Coherence Tomography Angiography (OCTA)

**DOI:** 10.7759/cureus.93077

**Published:** 2025-09-23

**Authors:** Anastasia Gkiala, Georgios Bontzos, Aikaterini Nikiforou, Georgios Smoustopoulos, Evgenia P Kontou, Ilias Gkizis, Christina Garnavou-Xirou, Tina Xirou

**Affiliations:** 1 Ophthalmology, Leicester Royal Infirmary, Leicester, GBR; 2 Ophthalmology, Korgialenio-Benakio General Hospital, Athens, GRC

**Keywords:** foveal avascular zone (faz), optical coherence tomography angiography (octa), pars plana vitrectomy, retinal detachment surgery, rhegmatogenous retinal detachment (rrd)

## Abstract

Purpose

Rhegmatogenous retinal detachment (RRD) occurs when the sensory retina separates from the retinal pigment epithelium (RPE) due to a retinal break. Pars plana vitrectomy (PPV) with gas or silicone oil tamponade is the primary surgical approach for RRD repair. This study evaluates microvascular changes in eyes undergoing PPV for RRD using optical coherence tomography angiography (OCTA) and compares outcomes between macula-on and macula-off cases.

Methods

This retrospective study included 45 patients (21 macula-on, 24 macula-off) who underwent RRD repair. OCTA was used to assess central retinal thickness (CRT), vessel density in the superficial (sVD) and deep capillary plexus (dVD), and the foveal avascular zone (FAZ). Comparisons were made between affected and fellow eyes, as well as between macula-on and macula-off cases.

Results

CRT was significantly different between macula-on and macula-off groups (p < 0.01). A weak but significant correlation was found between best-corrected visual acuity (BCVA) and FAZ area (r = 0.21, p = 0.018). Inferior detachments had lower dVD (32.16 ± 5.22%) than other quadrants (p < 0.05). dVD was reduced in affected eyes compared to fellow eyes in both macula-on (p = 0.01) and macula-off groups (p < 0.01), while sVD decreased significantly only in macula-off cases (p = 0.03).

Conclusion

RRD repair significantly impacts retinal microvasculature, particularly dVD reduction in macula-off cases and inferior RRDs. The correlation between FAZ enlargement and BCVA suggests microvascular alterations influence visual recovery. These findings highlight the need for further investigation into vascular recovery strategies after RRD repair.

## Introduction

Rhegmatogenous retinal detachment (RRD) is characterized by the separation of the sensory retina from the retinal pigment epithelium (RPE) due to a retinal break, permitting liquefied vitreous to enter the subretinal space and split the retina into two layers [1]. When the detachment involves the macular area of the retina, it is described as a ‘macula-off’ retinal detachment (RD) and is usually associated with poorer visual outcomes compared to ‘macula-on’ RD, where the central area remains intact. In a recent study, a panel of vitreoretinal experts reported a percentage of macula-off RDs at 58% upon patient presentation [2].

Management of an RRD involves surgery, the urgency of which is typically determined by the presence or not of a detached macula, the former being treated less urgently than the latter, which should ideally undergo surgery within 24 hours [3]. The rationale behind the surgical timeframe is determined by the time at which irreversible hypoxic damage is established in the detached retina. This includes the effect of RPE detachment but also changes in the retinal microcirculation. Experimental studies in models and humans have shown that photoreceptor death is induced as early as 12 hours and peaks around two to three days after RD [4].

Pars plana vitrectomy combined with either gas or silicone oil tamponade remains the primary treatment for RRD, though pneumatic retinopexy and scleral buckling may be considered in selected cases [5]. The reported success rates of these procedures are high, 81% for the former and >90% in the other two, and greatly depend on preoperative macula status and successful reattachment of the retina postoperatively [6]. However, despite a successful anatomical reattachment of the retina and even in the absence of macular involvement, the final visual outcome of some patients is still unsatisfactory [7].

Any type of retinal disease, such as retinal vein occlusion (RVO) and diabetic retinopathy (DR), but also RD, can cause changes in the retinal microvasculature. These changes can be detected by imaging modalities, such as fundus fluorescein angiography, a detailed but invasive imaging option, as well as optical coherence tomography angiography (OCTA), which is more easily attained, non-invasive, and associated with far fewer side effects [8].

The aim of this study is to present the visual as well as the microvascular changes in eyes that have undergone PPV repair for RRD. The fellow eye was used as a reference, while the location of the RRD was also taken into consideration.

## Materials and methods

This retrospective study received prior approval from the Institutional Review Board (IRB) of our institution (IRB of Korgialenio Benakio General Hospital, approval number 22835, dated September 5, 2023) and was conducted in accordance with the principles of the Declaration of Helsinki. All participants were informed about the study’s purpose, and written informed consent was obtained.

The fellow eye was used as a reference. The recruited patients did not have any ocular pathology in the fellow eye, including previous surgeries, apart from cataract extraction. Subjects with pathologic myopia (>6D), aphakia, history of intraocular inflammation, giant retinal tears, precipitating ocular trauma, or dense vitreous hemorrhage (2+ or greater) were also excluded.

The type of retinal detachment was categorized according to the affected quadrant for the purpose of this analysis (superior, inferior, temporal, nasal). This was determined by the site of the detached retina and not the site of the break. If more than one quadrant was affected, the most affected quadrant was included in the analysis. In cases where more than one quadrant was involved, the retinal detachment was labeled after the part where detachment was observed closer to the macula.

OCTA imaging was performed with the RTVue XR Avanti system (Optovue Inc., Fremont, CA, USA), operating at 70,000 A-scans per second with an 840 nm light source. Standard 3.0-mm and 6.0-mm HD scan modes were used to acquire 3.0 × 3.0 mm and 6.0 × 6.0 mm macular images centered on the foveola. The foveal avascular zone (FAZ) area was quantified on 3.0 × 3.0 mm scans using the automated AngioVue 2.0 software (Optovue Inc., Fremont, CA, USA). In cases of segmentation errors, FAZ boundaries were manually corrected with the built-in editing tools. All FAZ measurements were obtained from fovea-centered scans; quadrant classification in this study referred exclusively to the predominant detachment location, not to regional macular divisions.

Retinal thickness mapping (CRT, μm) and structural layer assessment were conducted using retina map mode. Segmentation errors caused by macular pathology were corrected manually with boundary editing and propagation tools. Images with scan quality <7/10 (software scale) were excluded. Manual adjustments were independently performed by two experienced graders (GS, CGX).

Vessel density analysis was performed on 6.0 × 6.0 mm HD scans, expressed as the percentage of area occupied by vessels in the macular region. Both superficial and deep vascular plexus densities were evaluated.

Statistical analysis was conducted using IBM SPSS Statistics for Windows, Version 22.0, Armonk, NY, USA. Data is reported as mean ± SD. Normality was tested with the Shapiro-Wilk test. Group differences were assessed with one-way ANOVA followed by Tukey’s post hoc test, using a two-sided significance level of α = 0.05. Post hoc power analysis (G*power 3.1.9.2, Heinrich-Heine-Universität Düsseldorf, Germany) indicated 0.97 power for an independent samples t-test with 45 subjects per group, effect size d = 0.75, α = 0.05, and 5% detectable difference in vascular density. Graphical analyses were generated in GraphPad Prism (GraphPad Software, Boston, MA, USA). Associations between FAZ area and final visual acuity were assessed using linear regression and Pearson correlation coefficients.

## Results

This study included 45 patients (62.2% female, n=28) with primary uncomplicated RRD. No history or clinical signs of prior macular pathology (e.g., cystoid macular edema (CME), epiretinal membrane (ERM), macular hole) were reported or documented for any of the included eyes, based on surgical records and clinical evaluation. Of these 45 eyes, 21 eyes had macula-on retinal detachment (46.7%) upon presentation, and 24 eyes had macula-off (53.3%). None of the patients had bilateral eye involvement. Surgery was performed within 48 h of diagnosis. All eyes underwent pars plana vitrectomy (PPV) with either gas (23G, C3F8) or silicone oil tamponade. In cases where silicone oil was used, we allowed three months after its removal before measurements were performed. The period of measurements following the RRD repair was at the six-month timepoint (Table 1). Two scans were obtained, from which the best quality was used for analysis.

**Table 1:** Baseline demographic and clinical characteristics of the study cohort

Characteristic	Total (n = 45)	Macula-on (n = 21)	Macula-off (n = 24)
Age, years (mean ± SD)	61 ± 12	59 ± 11	63 ± 13
Sex, n (%)	Male: 17 (37.8%)	Male: 8 (38.1%)	Male: 9 (37.5%)
	Female: 28 (62.2%)	Female: 13 (61.9%)	Female: 15 (62.5%)
Detachment location, n (%)	Superior: 18 (40%)	Superior: 8 (38%)	Superior: 10 (42%)
	Temporal: 11 (24%)	Temporal: 5 (24%)	Temporal: 6 (25%)
	Nasal: 8 (18%)	Nasal: 4 (19%)	Nasal: 4 (17%)
	Inferior: 8 (18%)	Inferior: 4 (19%)	Inferior: 4 (17%)
Tamponade used, n (%)	Gas: 37 (82%)	Gas: 18 (86%)	Gas: 19 (79%)
	Silicone oil: 8 (18%)	Silicone oil: 3 (14%)	Silicone oil: 5 (21%)

The central retinal thickness (CRT) of the participants was 289.58 ± 60.34 in the RD eye and 294.62 ± 59.41 in the fellow eye (p=0.15). However, it was measured 271.37 ± 52.78 in the mac-off group and 296.71 ± 37.81 in the mac-on group (p<0.01). The FAZ area was measured 0.28 ± 0.18 in the RD eyes, compared to 0.19 ± 0.12 in the fellow eyes (p=0.31) (Table 2). No significant difference was observed after accounting for macula-off status (p=0.19).

**Table 2:** OCTA characteristics according to macula status CRT: central retinal thickness; dVD: deep vessel density; OCTA: optical coherence tomography angiography

Characteristic	Macula-on (n=21)	Macula- off (n=24)	p value
CRT (µm)	296.71 ± 37.81	271.37 ± 52.78	p<0.01
dVD (%)	39.12 ± 3.84	36.74 ± 4.51	p < 0.01

**Figure 1:**
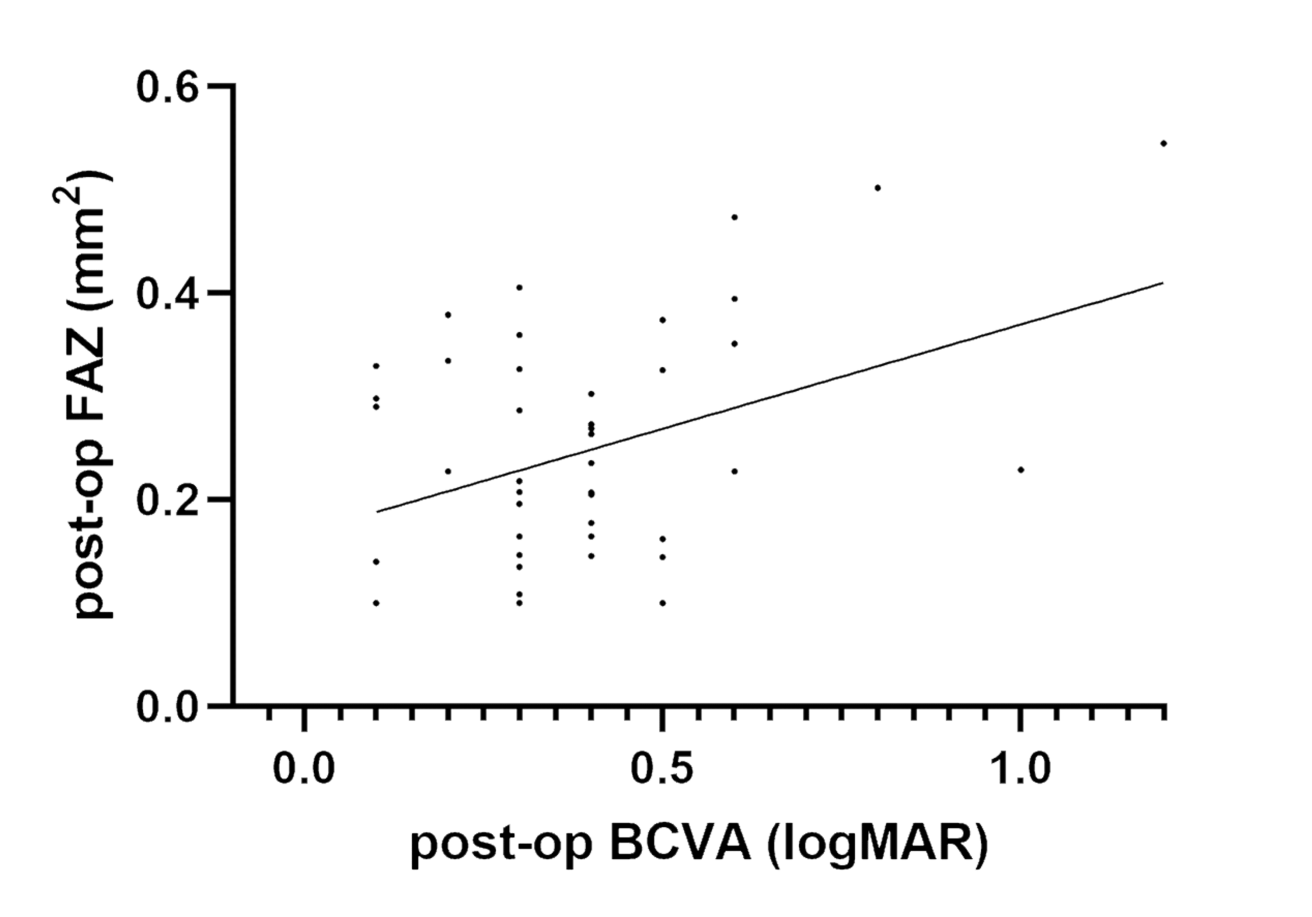
Weak but statistically significant correlation between BCVA and FAZ BCVA: best-corrected visual acuity; FAZ: foveal avascular zone; logMAR: logarithm of the minimum angle of resolution

Pearson correlation analysis demonstrated a weak but statistically significant correlation between best-corrected visual acuity (BCVA) and FAZ area (r = 0.21, p = 0.018), suggesting a weak link between FAZ expansion and diminished visual acuity (Figure 1).

Mac-off detachment cases had a significantly lower deep vessel density (36.74 ± 4.51%) compared to mac-on detachments (39.12 ± 3.84%) (p < 0.01) (Table 2).

**Figure 2:**
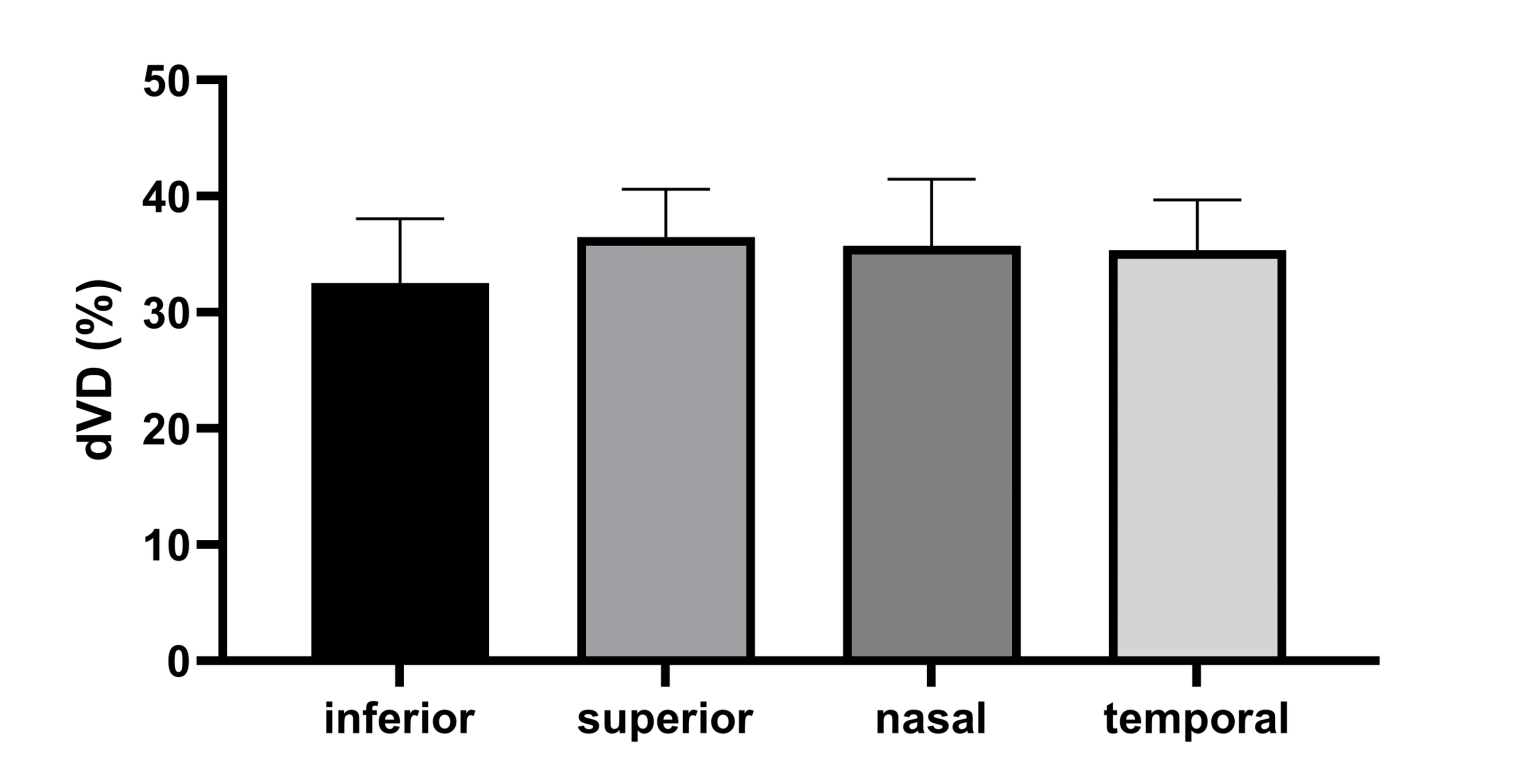
Statistically significant reduction in deep vessel density in eyes with inferior retinal detachment dVD: deep vessel density

One-way ANOVA revealed a statistically significant difference in deep vessel density (dVD) across the four detachment locations (F(3,41) = 3.85, p < 0.05). Post-hoc analysis using Tukey’s HSD test indicated that eyes with inferior detachment showed a modest but statistically significant reduction in deep vessel density (32.16 ± 5.22%) compared to superior, temporal, and nasal detachments (p < 0.05) (Figure 2). For superficial vessel density (sVD), the one-way ANOVA did not reveal a statistically significant difference across the four detachment locations (F(3,41) = 2.1, p = 0.08), suggesting no substantial variation in this measure based on detachment location.

When comparing fellow eye dVD to the RRD dVD, the analysis revealed a significant difference in the macula-on subgroup (p = 0.01) and a highly significant difference in the macula-off subgroup (p < 0.01). Similarly, a comparison of fellow superficial density sVD with the RRD sVD showed an insignificant difference in the macula-on group (p = 0.61) but a significant difference in the macula-off group (p = 0.03), highlighting a potentially distinct impact on sVD in macula-off detachments.

## Discussion

Vascular alterations in RRD have been reported throughout the literature, and retinal perfusion is known to be impaired for several years [9]. Recent technological advances have rendered OCTA an important part of the clinical routine, used to determine vascular abnormalities in retinal disease [10]. The most commonly tested parameters that describe the status of the retinal vasculature on OCTA images are the FAZ, the vessel density in the deep and superficial retinal plexus, and the CRT. The deep and superficial retinal plexus constitute the macular perfusion system, which is responsible for meeting the metabolic needs of the neurons. Any significant reduction in vessel density could indicate retinal ischemia [11]. CRT has also been reported to correlate with visual quality [12]. The FAZ is the physiologically avascular area in the center of the macula (fovea), which is responsible for central vision.

FAZ size does not usually correlate with visual function in healthy eyes but can be of visual significance in the context of retinal pathology [13,14]. In our work, we found that, FAZ area has a weak but significant correlation with final visual acuity (Figure 1). Current literature presents debatable opinions on how FAZ is affected in eyes with RD repair. Previous research, by Woo et al. showed that both the post-operative superficial and deep FAZ area increased in size in all the mac-off eyes included in the study when compared to the mac-on eyes [15]. The changes in the FAZ zone correlated with visual acuity. These findings were further supported by Agarwal et al. and Chatziralli et al. [16,17]. However, these results could not be confirmed by other researchers [18,19].

Similarly, visual density (VD), while seemingly impaired after an operated RRD, does not follow a uniform trend across literature sources [6]. A previous systematic review and meta-analysis of seven studies analyzing retinal microcirculation using OCTA indicated that sVD was not significantly affected when operated eyes were compared with their fellow, whereas dVD was significantly increased even after reattachment of the retina, when compared with control eyes [6]. However, other research teams did not find significant differences in any plexus across macula-on and macula-off cases [20,21].

The conflicting findings could stem from variations in the control groups used in each study. The sVD is closely linked to retinal arterioles and may experience higher perfusion pressure compared to dVD, which primarily consists of venous collecting channels. As a result, the dVD might be inherently more susceptible to increases in venous pressure. Additionally, the dVD is situated in a watershed-like region, where oxygen saturation tends to be lower than in both the inner and outer retina. When retinal detachment occurs, oxygen supply from the choriocapillaris to the dVD can be significantly restricted by the presence of subretinal fluid [15].

The selection of the healthy fellow eye as control is considered to be more appropriate, given the comparable FAZ and vessel density in the same patients, but its variation among different individuals should still be taken into account [22].

Our study did not confirm an increased FAZ size in eyes with repaired mac off retinal detachment compared to controls. There was a weak but significant correlation between FAZ and BCVA in the logarithm of the minimum angle of resolution (logMAR), indicating that increased FAZ is associated with reduced visual acuity. This correlation has also been supported by other studies, such as those by Woo et al., Bonfiglio et al., and Christou et al. [15,18,23]. Despite this, the modest effect size suggests that FAZ enlargement alone is unlikely to serve as a strong predictor of postoperative visual outcome.

In our study patients, we allowed adequate time for the neurosensory retina to heal, following its reattachment to the RPE. It has been suggested that in cases of retinal detachment, there is double hypoxic damage, both from detachment of the neurosensory retina from the pigment epithelium, but also from changes in the retinal circulation and an increase in the concentration of inflammatory mediators. This can cause irreversible damage that cannot be restored even after successful retinal reattachment [15]. Wang et al. retrospectively studied patients with mac-off RRD for 12 weeks post-repair and concluded that flow density in sVD, dVD, and choriocapillaris gradually increased over time but never reached levels of the fellow eye, indicating gradual but not complete recovery of macular perfusion [24]. This incomplete recovery has been linked to possible macular hypoxia. Flow in sVD and dVD was not correlated with final BCVA [24]. Interestingly, in this study, researchers showed that the choriocapillaris perfusion recovery reached a plateau just before the recovery of the sVD and dVD circulation [24]. This indicates a different recovery speed of choroidal and retinal circulation, and the fact that a restored choroidal circulation might be a prerequisite for retinal circulation restoration and vision improvement. Further research supports the fact that restoration of microcirculation in the affected retina requires a minimum of six months or even more time to be achieved after successful surgery [15]. In another context, Resch et al. reported changes in the microvasculature in patients who had undergone RRD repair more than two years ago [25]. The authors suggest that the deep and parafoveal region is more affected in the earlier postoperative time, while the superficial, foveal region, and the extent of the non-flow area are more affected later in time.

When comparing macula-on and macula-off cases, previous knowledge suggests that vessel density is uniformly impaired significantly in superficial and deep plexuses for detached maculas [19,24,26], while results are more controversial for macula-on cases [18,27]. This indicates that macula detachment could have a significant role in microvasculature changes in RRD eyes [18,27]. Another finding of our study is that CRT was significantly. reduced in eyes with macula off RRD compared to macula on, which is also supported by other researchers [18,28]. It should be noted that reduced CRT could also interfere with reported findings of reduced VD.

Neural apoptosis is known to be associated with reduced vessel density parameters. For example, patients with geographic atrophy (GA) due to age-related macular degeneration (AMD) demonstrate a significant decrease in retinal vessel densities in all retinal plexuses compared to normal eyes [29]. This has been attributed to loss of photoreceptors and ganglion cells or to potential increased oxygen flow from choroid to inner retina, causing vasoconstriction [30]. These results underline that areas of macular atrophy may act as a potential artifact to the measured vascular density.

In our study, we also attempted to compare our results among four different RRD locations. We found that inferior RRDs display significantly lower values dVD (Figure 2). A possible explanation for the reduced deep VD in cases with inferior retinal detachment might be the fact that inferior retinal detachments carry a lower risk and rate of progression and involve the macula area and thus causing significant vision reduction. Therefore, they can become chronic more easily than retinal detachments in other locations. In addition, chronic retinal detachments carry a higher risk of complications, such as proliferative vitreoretinopathy (PVR), which can further compromise vascular restoration.

Our study has certain limitations that should be acknowledged. Firstly, the sample size, while sufficient for statistical power, remains relatively small, limiting the generalizability of our findings. Larger, multicenter studies with more diverse patient populations would help validate our results. Secondly, the timing of OCTA imaging postoperatively was standardized to six months, but the vascular recovery process may extend beyond this period, potentially affecting vessel density measurements. Longitudinal studies with multiple follow-up time points would provide better insight into the progression of microvascular changes over time. Finally, the presence of subclinical ischemic changes in the fellow eyes, which could have influenced our comparisons, was not assessed. Lastly, despite the advantages of OCTA as a non-invasive imaging tool, segmentation errors and image artifacts remain a concern, particularly in cases with structural retinal changes post-RRD repair. Future studies integrating OCTA findings with functional visual outcomes and histopathological data would further elucidate the clinical significance of these vascular alterations.

## Conclusions

This study highlights the impact of RRD repair on retinal microvasculature as assessed by OCTA, particularly the differential effects observed in macula-on versus macula-off cases. Our findings suggest that macular detachment significantly influences dVD, with macula-off cases demonstrating a greater reduction in perfusion. Additionally, inferior retinal detachments were associated with significantly lower dVD, possibly due to chronicity and a higher risk of PVR. Finally, the observed correlation between FAZ enlargement and reduced visual acuity could further support the role of microvascular alterations in postoperative visual outcomes.
